# Technological Methods for Reducing the Content of Fructan in Wheat Bread

**DOI:** 10.3390/foods8120663

**Published:** 2019-12-10

**Authors:** Ewa Pejcz, Radosław Spychaj, Zygmunt Gil

**Affiliations:** Department of Fermentation and Cereals Technology, Faculty of Biotechnology and Food Science, Wrocław University of Environmental and Life Sciences, ul. Chełmońskiego 37, 51‐630 Wrocław, Poland; radoslaw.spychaj@upwr.edu.pl (R.S.); zygmunt.gil@upwr.edu.pl (Z.G.)

**Keywords:** wheat bread, fructans, irritable bowel syndrome (IBS), fermenting oligosaccharides, disaccharides, monosaccharides, and polyols (FODMAPs)

## Abstract

Irritable bowel syndrome (IBS) is a functional disorder of the gastrointestinal system. Adherence to a low-FODMAP (fermenting oligosaccharides, disaccharides, monosaccharides, and polyols) diet may be one of the solutions in this case. The major FODMAP carbohydrates found in wheat bread are fructans. The objective of this study was to produce wheat bread with a reduced fructans content. Breads were made from light and whole grain flour obtained from common wheat using two methods of dough development—I-stage method with the use of yeast, and II-stage method with the use of yeast and sourdough with a pure culture of *Lactobacillus plantarum*. Four different fermentation times were tested—60, 90, 120, and 150 min. Afterwards, quality attributes (loaf volume, crust and crumb color, and sensory properties) of the produced breads were evaluated, and the fructans content was determined. The results demonstrated that all the factors influenced the quality of wheat breads, as well as their fructans content. Breads made with the II-stage method and light flour had a lower content of fructans, which was decreased in breads along with extending the time of dough fermentation. The greatest impact on fructans content decrease in wheat bread was ascribed to the use of light flour, the II-stage method of dough development coupled with a dough fermentation time prolongation to 150 min.

## 1. Introduction

Irritable bowel syndrome (IBS) is a functional disorder of the gastrointestinal tract that may afflict up to 12% of the European population [1,2]. Its symptoms include recurring abdominal pain and discomfort, constipations, altered stool consistency, and variable defecation rhythm [3].

Many works have shown a diet with a low content of short-chain carbohydrates to help in alleviating IBS symptoms [4,5]. A group of these compounds has been named FODMAPs, which means—fermenting oligosaccharides, disaccharides, monosaccharides, and polyols. It contains carbohydrates and carbohydrate-derived alcohols that are poorly absorbable in the small intestine and therefore, fermented in the subsequent segments of the gastrointestinal tract where they serve as food to enteric bacteria. So far, FODMAP oligosaccharides have been known for their positive impact on a human body. Fructans represent the soluble fraction of dietary fiber, hence they exert a prebiotic effect. In addition, they affect the lipid profile and cause a reduction in the serum levels of cholesterol.

The major fermenting oligosaccharides include fructooligosaccharides (fructans) and galactooligosaccharides (GOS). Fructans are low-molecular-weight, often branched polymers of D-fructose. They are non-digestible carbohydrates and display various nutritional properties which affect metabolism of microorganisms, absorption of minerals, and sensation of satiety. They may be found in various plant-based food products, with wheat being one of their important sources [6]. In turn, GOS are compounds composed of a few galactose molecules connected to a lactose molecule with α-1, 4; β-1, 6 glycosidic bonds. They are natural components of milk, but also of certain fruits and vegetables [7].

Fructans are the predominant FODMAP carbohydrate in wheat bread. Considering their high content in wheat bread, especially whole meal bread (1.4–1.7 g/100 g d.m. in wheat flour and 0.7–2.9 g/100 g d.m. in whole grain wheat flour), this type of bread is not recommended in the low-FODMAP diet [8].

Despite a substantial decrease in the consumption of bread, it is still the basic element of diet to many people. Wheat bread is a source of the fructooligosaccharides belonging to the FODMAP group. Its exclusion from everyday diet may pose some risks due to the reduced intake of dietary fiber being a substrate for enteric bacteria. In order to make the consumption of wheat bread possible to people suffering from ailments of the gastrointestinal system, attempts have been made to reduce its fructans content. Using a sourdough, which displays specific properties of FODMAP metabolism, in wheat bread production may contribute to a significant decrease in the FODMAP content in bread without reducing dietary fiber [9].

Wheat grain contains low monosaccharides. Its main saccharides include—saccharose, raffinose, and fructans. During sourdough fermentation, the amylase and glucoamylase of wheat flour degrade the damaged starch to maltose and glucose. The content of fructans is reduced in wheat bread to 1–1.5% upon the use of the I-stage method of dough development, in which all the dough ingredients are mixed together in one mixing stage before dough fermentation. Their degradation is mainly attributable to the microorganisms used in the fermentation process. The invertase activity of *Saccharomyces cerevisiae* causes partial hydrolysis of flour fructans. In the case of dough made with the addition of *S. cerevisiae*, the rate of fructans degradation decreases along with their chain length increase [6].

The inclusion of specific cereal-adapted lactic acid bacteria extends the metabolic capability of fermentative microbiota. In turn, the extended time of fermentation significantly increases the contribution of flour enzymes in the conversion and degradation of dough components [10].

Fructans of cereals are partially degraded, however when a leavening agent is used, the released fructose is also partially converted into mannitol by *Lactobacillus* bacteria. Mannitol is a polyol which is rapidly fermented by enteric microflora and hence it is also classified as a FODMAP component. Therefore, its degradation in the production process of low-FODMAP bread requires the assistance of the mannitol-fermenting *Lactobacillus* bacteria. Mannitol metabolism in *Lactobacillus* bacteria is mediated by a mannitol-specific phosphotransferase system PTS. The enzymes participating in mannitol conversion are secreted by homofermentative bacteria from the following genera—*Lactobacillus L. delbrueckii L. casei L. plantarum*, and *L. salivarius* [9].

The use of sourdough, which displays specific metabolic properties, in wheat bread production may help in significantly reducing the FODMAP content in bread [11]. The low-fructan wheat bread may represent a food product intended for people with diagnosed intolerance to fructans—as a major group of the FODMAP component in bread. Considering the above, an attempt was undertaken in this study to produce wheat bread with a reduced content of fructans.

## 2. Materials and Methods

The experimental material included the grain of common wheat that was ground in a Quadrumat Sernior laboratory mill (Brabender, Duisburg, Germany) into—whole grain flour and light flour. In order to obtain the whole grain flour, all the side products of milling (bran and shorts) were grinded in a Hagberg-Perter mill and mixed with flour. The determination of the ash content of the flours also allowed for differentiating them into flour types: light flour—type 450, and whole grain flour—1650-type.

The flours produced were analyzed for—fraction size, ash content with the AOAC Method 930.05 [12] method, and protein content with the Kiejdahl method according to AACC Method 46-12.01 [13] using the Kjeltec 2400/2460 apparatus. Doughs made of the flours examined were determined for rheological parameters, including water absorption, development time, stability, softening, and maximum viscosity, using a Mixolab apparatus (Chopin Technologies, Villeneuve-la-Garenne, France) according to AACC Method 54-60.01 [14].

The dough for bread making was prepared according to the following formula: flour—100%, water—in the amount needed to produce dough with a consistency of 300 FU, fresh commercial *S. cerevisae* yeast—3.5%, and salt—1%. In the I-stage method, the dough was made by mixing its ingredients, placed in molds, and placed directly to the fermentation chamber. In the II-stage method, the first stage was I-type sourdough of 1:1 flour to water ratio with the addition of a pure culture of *L. plantarum* (type strain 20174), which was left to ferment at a temperature of 30 °C and humidity of 85% for 24 h before use. The second stage dough consisted of the sourdough in the amount accounted for 50% of flour and the remaining flour, water, yeast, and salt. The dough was mixed in a fast-rotating laboratory mixer (Stephan, Hameln, Germany), then portioned into four bites of equal sizes, placed in pans, transferred to a fermentation chamber (IBIS, Szubin, Poland), and fermented for 60, 90, 120, and 150 min. After one hour of the fermentation process (except for the first process, after which the bites were directly intended for baking), the bites were kneaded every 30 min, while the final fermentation lasted till the bites reached oven maturity. The doughs were baked into breads in an IBIS GT 600 oven (IBIS, Szubin, Poland) at a temperature of 220 °C for 30 min. The samples were made in all possible combinations—both types of flours were tested in both dough development methods, and for each dough, four different fermentation times were performed.

After baking, the bread volume was measured with a SA-WY apparatus (ZBPP, Bydgoszcz, Poland) and expressed as per 100 g of bread. The color of the bread crust and crumb was measured with a Minolta Chroma Meter CR 410 (Konica Minolta, Tokyo, Japan). The bread samples were also subjected to the organoleptic evaluation in a 9-point hedonic scale to assess for the following quality attributes—appearance, crust color, crumb color, consistency, aroma, and taste. The organoleptic panel consisted of 10 semi-trained food technology master’s students (women and men) between 22 and 25 years old. The bread samples were served at room temperature.

The lyophilized and disintegrated samples of breads were analyzed in two replicates for the content of fructans with a fructan determination kit (Megazyme, Bray, Ireland), based on the enzymatic removal of interfering sugars contained in the sample (e.g., starch), followed by measurement of the fructan content by a colorimetric measurement at 410 nm after enzymatic conversion into glucose and fructose [15].

The traits of the flours were compared with the one-way analysis of variance and Duncan’s test at α = 0.05. In turn, the multi-way analysis of variance and Duncan’s test were used to compare the effects of dough development method, fermentation time, and flour type on the quality traits and fructans content of wheat breads, at α = 0.05. All statistical analyses were performed using Statistica software version 13.3 (StatSoft Polska Sp. z o.o., Kraków, Poland).

## 3. Results and Discussion

Wheat flour was evaluated considering the following parameters—fraction size, ash content, protein content, fructan content, water absorption, dough development time, dough stability, dough softness, and maximum viscosity (Table 1).

The 1650-type flour was characterized by a greater fraction size (127.98) than the 450-type flour (95.42). The ash content of the flours was a factor which differentiated them into types; it reached 0.45% in the light flour and 1.65% in the whole grain flour. The 1650-type flour had a higher protein content (11.72 g/100 g) than the 450-type flour (10.10 g/100 g). The content of fructans was higher in the 1650-type flour (1.6 g/100 g than in the 450-type flour (1.2 g/100 g). This is consistent with findings reported by Praznik et al. [16], who determined the fructans content in wheat whole grain at 1.62 g/100 g. A similar result was achieved by Haska et al. [17], who reported the fructans content in wheat flour at 1.60 g/100 g. In turn, Jasińska-Kuligowska et al. [18] analyzed the content of fructans in grain milling products and showed it was the highest in the bran, which indicates that fructans are accumulated in the outer parts of the grain.

The dough Mixolab rheological parameters are presented in Table 2. The 1650-type flour displayed a higher water absorption (55.25%) compared to the 450-type flour (45.10%). The dough development time was significantly higher in the case of dough made of the 1650-type flour (3.7 min) compared to dough made of the 450-type flour (1.53 min). Dough made of the 1650-type flour was also characterized by greater stability than that prepared from the 450-type flour (8.89 min and 7.96 min, respectively). Dough softening was greater in the dough prepared from the 1650-type than from the 450-type flour. Light flour was also characterized by higher amylolytic activity and starch retrogradation than whole grain flour.

The quality assessment of wheat bread was carried out 24 h after baking. The assessment included the following attributes—volume/100 g bread, as well as L*, a*, and b* color parameters of the crust and crumb (Table 3). The flour type, dough development time, and dough development method influenced the bread loaf volume. Breads made with the I-stage method had a higher volume (336 cm^3^/100 g bread) than these produced with the II-stage method (287 cm^3^/100 g bread). However, *L. plantarum* containing sourdough addition had no effect on the loaf volume of breads made by other researchers [19,20]. A higher loaf volume was determined for breads made of the 450-type flour (332 cm^3^/100 g bread) than for these made of the 1650-type flour (290 cm^3^/100 g bread).

The crust color was affected by the flour type as well as the dough development method and dough fermentation time. The color of the crust of breads made of the 450-type flour was significantly lighter (L* = 51.88) than that of the breads made of the 1650-type flour (L* = 39.59). Breads produced with the II-stage method had lighter crust (L* = 47.62) than these produced with the I-stage method (L* = 43.84). Along with the progressing time of dough fermentation, the crust of the breads was also lighter (L* = 43.94 for fermentation time of 60 min, L* = 48.14 for fermentation time of 150 min). The crust color of breads produced from the 450-type flour was more yellow (b* = 27.23) than that of the breads produced from the 1650-type flour (b* = 16.92). The crumb color was influenced by the dough fermentation time, flour type, and dough development method. The crumb of breads made of the 450-type flour was significantly lighter (L* = 70.97) than the crumb of breads made of the 1650-type flour (L* = 47.47). The bread crumb turned darker along with the progressing time of dough fermentation (L* = 60.54 for 60 min, L* = 57.61 for 150 min). The value of color parameter a* determined for the crumb of breads made of the 450-type flour indicates a greater contribution of the green color (a* = −3.65), and for breads made of the 1650-type flour it indicates a greater contribution of the red color (a* = 3.95). The II-stage method of dough development contributed to a more reddish color (a* = 0.31) of the bread crumbs compared to the I-stage method (a* = −0.01). The crumb of breads prepared from the 450-type flour was characterized by a significantly higher contribution of the yellow color (b* = 22.53) compared to the crumb of breads made of the 1650-type flour (b* = 15.74). The crumb of breads baked from doughs fermenting for 60 min was significantly more yellow (b* = 20.36) compared to breads made of doughs allowed to ferment for 90, 120, and 150 min. The bread color was determined by flour type—breads made of the whole grain flour were darker due to the bran contribution in the flour. The bread crumb color turned darker along with dough fermentation time extension. During the fermentation process, starch is hydrolyzed to molecules of maltose and glucose, which are representatives of reducing sugars. The longer the fermentation time, the higher the content of reducing sugars in the dough, which undergo caramelization during baking and, therefore, cause a darker color of bread.

**Figure 1:**
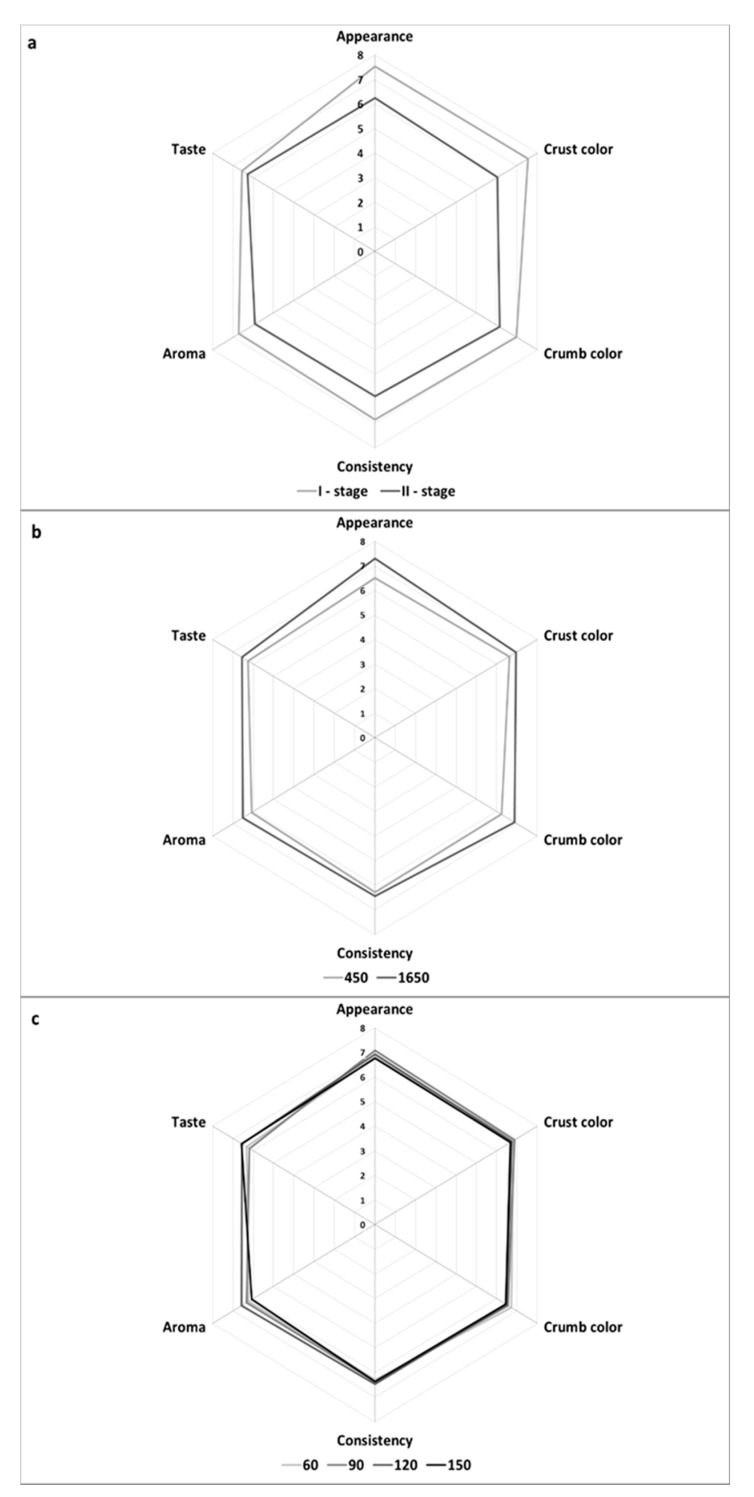
Sensory evaluation of wheat bread depending on dough development method (**a**), flour type (**b**) and dough fermentation time (**c**) in a 9-point hedonic.

Bread was also subjected to the organoleptic evaluation (Figure 1). The organoleptic traits of wheat bread were affected to the greatest extent by the dough development method, whereas the flour type turned out to be of lesser significance in this case. In contrast, the dough fermentation time had no effect on the organoleptic traits of the analyzed wheat breads.

The breads made of the 450-type flour were scored significantly higher for general appearance than these made of the 1650-type flour. The breads obtained from dough developed with the I-stage method also scored significantly higher for this attribute than the breads made of dough developed with the II-stage method. The crust color of breads produced from dough developed with the I-stage method was evaluated as more desirable than that of breads made from dough developed with the II-stage method. The crumb color scored higher in the case of breads made of the 1650-type than of 450-type flour. The crumb color of breads made of dough developed with the I-stage method received higher scores than that of breads from dough developed with the II-stage method. The consistency of breads baked from dough developed with the I-stage method was scored higher than that of breads made of dough developed with the II-stage method. The aroma of breads was also more desirable in those made of doughs developed with the I-stage than with the II-stage method. In contrary, the results of Robert et al. [20], Faqir et al. [21] and Jayaram et al. [22] showed better sensory properties of breads with sourdough addition comparing to only yeast-fermented samples.

**Figure 2:**
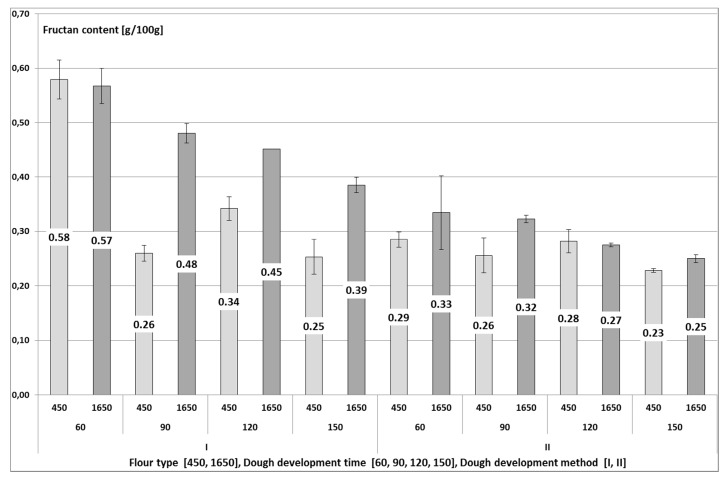
The content of fructans (g/100 g d.m.) in wheat bread of different flour type (F), dough development method (M) and dough fermentation time (T). Values represent the means of two replicates. Least Significant Differences LSD_F_ = 0.03, LSD_T_ = 0.04, LSD_M_ = 0.03, LSD_FxT_ = 0.06, LSD_FxM_ = 0.04, LSD_TxM_ = 0.06, LSD_FxTxM_ = 0.08.

The content of fructans in the produced wheat breads was affected by the dough development method, dough fermentation time, and flour type (Figure 2). The breads made of the 1650-type flour had a higher content of fructans than the breads made of the 450-type flour. This was due to the fact that fructans are concentrated in the outer layers of a kernel, and that the 1650-type flour was the whole grain flour. Biesiekierski et al. [23] compared the fructans content in light bread (made of light flour) and dark bread (made of whole grain flour), and found a higher content of fructans in the dark bread (1.42 g/100 g) than in the light bread (1.05 g/100 g). When comparing breads made of light and whole grain flours, Whelan et al. [6] showed no significant difference in the fructans content, which reached 0.86 g/100 g and 0.88 g/100 g, respectively.

The II-stage method of dough development caused a 33% lower content of fructans in the breads compared to the control sample. Fraberger et al. [24] analyzed 13 strains of microorganisms for their fructans-reducing capability. In each case, the metabolism of the microbiota led to a significant reduction of fructans in bread, even by 83%, compared to the control bread. Andersson et al. [25] compared the content of fructans in breads made of dough which was fermented with the addition of yeast and bacteria or yeast alone. They found a significantly lower content of fructans in bread produced with the addition of bacteria and yeast. In our study, the fructans content was 33% lower in bread produced with the II-stage method (type II sourdough with the addition of a pure culture of *L. plantarum*) than in the control bread.

With elongation of dough fermentation time, the mean content of fructans decreased from 0.44 g/100 g (60 min) to 0.28 g/100 g (150 min). A similar effect was achieved in a study by Struyf et al. [8] where after 60 min of dough fermentation, the content of fructans in the wheat bread decreased by 45%, whereas in bread made of dough allowed to ferment for another hour, it decreased by 32%. Menezes et al. [26] claims that short fermentation lasting 30–180 min causes impaired hydrolysis of FODMAP components. The results of Faqir et al. [21] show decreasing contents of saccharides with the elongation of fermentation time. Fermentable carbohydrates (saccharose, maltose, glucose, and fructose) are rapidly depleted in the first hours of the fermentation process, whereas more time is needed for the hydrolysis of carbohydrates with a higher degree of polymerization (e.g., fructans).

The breads produced with the I-stage method had a higher content of fructans when made of the 1650-type (0.47 g/100 g) than of the 450-type (0.38 g/100 g) flour. The fructans content decreased in breads made of both flour types along with elongation of dough fermentation time. On average, 150-min of dough fermentation reduced the fructans content in bread by 37% compared to the shortest fermentation time tested (60 min). In the case of breads made of the 450-type flour, the content of fructans decreased rapidly when the dough was left to ferment for 90 min. In the case of breads made of the 1650-type flour, the fructans content decreased successively along with dough fermentation time elongation. The highest fructans content was determined upon the use of the I-stage method of dough development and the shortest dough fermentation time—60 min, i.e., 0.58 g/100 g in breads made of the 450-type flour and 0.57 g/100 g in breads made of the 1650-type flour. In contrast, the lowest fructans content was obtained in two types of bread, the first one—made of the 450-type flour with the II-stage method and dough fermentation time of 150 min (0.23 g/100 g fructans), and the other—made of the 1650-type flour with the II-stage method and dough fermentation time of 150 min (0.25 g/100 g fructans). These contents of fructans did not differ significantly. In the breads produced from the 450-type flour, the fructans content was reduced by 60%, whereas in the breads made of the 1650-type flour—by 56%, which shows their similar content in breads produced with II-stage method of dough development and with the longest time of dough fermentation. This indicates that the flour type was insignificant, because the content of fructans could be reduced in wheat bread by manipulating dough fermentation time and dough development method. It means that the most important factors affecting the fructans content in the production process of low-fructan breads are the dough development method (involving the use of yeast but also lactic acid bacteria being capable of metabolizing fructooligosaccharides) and dough fermentation time (extended to 150 min).

## 4. Conclusions

Fructans are the major group of FODMAP components in wheat bread. In order to produce wheat bread with a reduced content of fructans, we investigated the effect of the flour type, dough development method, and dough fermentation time, on their content in bread. Their reduction in bread was affected to the greatest extent by the use of the II-stage method of dough development consisting of the use of a sourdough with the addition of a pure culture of *L. plantarum*. A lower content of fructans was also obtained in the bread made of the 450-type flour than in bread produced from the 1650-type flour, as well as in breads made of dough allowed to ferment for the longest time examined. The greatest impact on the fructans content reduction in wheat bread was attributable to the simultaneous elongation of dough fermentation time to 150 min and use of the II-stage method of dough development. Flour type, dough fermentation time, and dough development method also had a significant effect on the quality of the wheat bread produced. The II-stage dough development method contributed to the deterioration of the sensory properties of wheat bread. Future investigations into the feasibility of producing low-FODMAP wheat bread should focus on the choice of bacterial strains used for sourdough production, on elongation of its fermentation time, and on the appropriate selection of dough development method to ensure a high quality of bread.

## Figures and Tables

**Table 1:** Quality parameters of wheat flour.

Flour Type	Fraction Size (µm)	Ash Content (g/100 g d.m.)	Protein Content (g/100 g d.m.)	Fructans Content (g/100 g d.m.)
450	95.41 ± 0.72 ^b^	0.45 ± 0.01 ^b^	10.07 ± 0.04 ^b^	1.18 ± 0.05 ^b^
1650	127.98 ± 3.81 ^a^	1.65 ± 0.02 ^a^	11.72 ± 0.02 ^a^	1.59 ± 0.07 ^a^

Values represent the means of two replicates. Small letters in the same column denote significant differences according to Duncan’s test (*p* ≤ 0.05).

**Table 2:** Mixolab parameters of wheat doughs.

Flour Type	Water Absorption (%)	Dough Development Time (min)	Dough Stability (min)	Dough Softening (C2) (Nm)	Peak Visosity (C3) (Nm)	Activity of Amylolytic Enzymes (C4) (Nm)	Retrogradation (C5) (Nm)
450	45.10 ± 0.00 ^b^	1.53 ± 0.05 ^b^	7.96 ± 0.25 ^b^	0.45 ± 0.01 ^b^	2.17 ± 0.00 ^a^	1.97 ± 0.00 ^a^	2.97 ± 0.01 ^a^
1650	55.25 ± 0.01 ^a^	3.70 ± 0.28 ^a^	8.89 ± 0.05 ^a^	0.57 ± 0.00 ^a^	1.99 ± 0.00 ^a^	1.11 ± 0.01 ^b^	1.82 ± 0.01 ^b^

Values represent the means of two replicates. Small letters in the same column denote significant differences according to Duncan’s test (*p* ≤ 0.05).

**Table 3:** Physical features of wheat bread depending on flour type, dough development method and dough fermentation time.

	Levels of Factor	Loaf Volume (cm^3^)	Crust Color			Crumb Color		
			L*	a*	b*	L*	a*	b*
Flour type	450	332 ± 113 ^a^	51.88 ± 6.90 ^a^	10.47 ± 6.94 ^a^	27.23 ± 3.23 ^a^	70.97 ± 2.44 ^a^	−3.65 ± 0.91 ^b^	22.53 ± 2.21 ^a^
	1650	290 ± 149 ^b^	39.59 ± 2.24 ^b^	9.47 ± 0.62 ^a^	16.92 ± 2.83 ^b^	47.47 ± 4.94 ^b^	3.95 ± 0.23 ^a^	15.74 ± 1.20 ^b^
Dough development method	I-stage	336 ± 71 ^a^	43.84 ± 7.95 ^b^	10.99 ± 6.69 ^a^	20.53 ± 7.44 ^a^	59.66 ± 12.41 ^a^	−0.01 ± 3.95 ^b^	18.90 ± 4.54 ^a^
	II-stage	287 ± 75 ^b^	47.62 ± 7.72 ^a^	8.95 ± 1.46 ^a^	23.62 ± 6.85 ^a^	58.78 ± 12.63 ^a^	0.31 ± 3.86 ^a^	19.41 ± 2.97 ^a^
Dough fermentation time	60	324 ± 113 ^a^	43.94 ± 7.79 ^b^	10.39 ± 0.78 ^a^	17.85 ± 6.17 ^a^	60.54 ± 10.67 ^a^	0.30 ± 3.95 ^a^	20.36 ± 4.57 ^a^
	90	302 ± 99 ^c^	43.95 ± 8.40 ^b^	9.80 ± 0.88 ^a^	20.70 ± 7.19 ^a^	60.37 ± 12.57 ^a^	0.08 ± 4.08 ^a^	19.19 ± 3.18 ^b^
	120	310 ± 172 ^b^	46.89 ± 7.49 ^a^	11.45 ± 9.58 ^a^	26.95 ± 7.01 ^a^	58.37 ± 11.46 ^b^	0.12 ± 3.85 ^a^	18.63 ± 3.82 ^b^
	150	309 ± 167 ^b^	46.14 ± 8.10 ^a^	8.23 ± 1.31 ^a^	22.80 ± 7.53 ^a^	51.61 ± 15.31 ^b^	0.12 ± 3.96 ^a^	18.34 ± 3.65 ^b^

Values represent the means of two (loaf volume) or five (color parameters) replicates. Small letters in the same column denote significant differences according to Duncan’s test (*p* ≤ 0.05).
